# Relation Between the Degree of Use of Smartphones and Negative Emotions in People With Visual Impairment

**DOI:** 10.3389/fpsyg.2021.653796

**Published:** 2021-05-10

**Authors:** Eun-Young Park

**Affiliations:** Department of Secondary Special Education, Jeonju University, Jeonju, South Korea

**Keywords:** smartphone, people with visual impairment, depression, anxiety, loneliness

## Abstract

The use of smartphones has become commonplace, even among people with disabilities. The purpose of this study was to investigate the effect of smartphone use on the negative emotions of people with visual impairment. This study analyzed data from 30 respondents with visual impairments obtained from the 2016 Internet Overdependence Survey in South Korea. The analysis was based on partial least squares regression with information search, leisure, communication, and online transactions as independent variables, and negative emotions comprising depression, anxiety, and loneliness as the dependent variables. Among people with visual impairment, the use of smartphones as a means of communication decreased negative emotions while their use for leisure or information search was related to an increase in negative emotions such as depression and loneliness. Use for information retrieval was found to be associated with a low level of anxiety, and use for online transactions was associated with low loneliness. The results of this study showed that the use of the Internet can be a means of providing interaction opportunities and reducing negation emotions for people with visual impairment.

## Introduction

According to a survey conducted in 2015, there are more than 1.5 billion smartphone users worldwide and, overall, more than 1 billion smartphones are estimated to have been sold (Demirci et al., 2015). Smartphone ownership was 56% in 2013 in the United States (Smith, 2013) and 79% in 2012 in Switzerland (Suter et al., 2015). Based on a survey conducted in South Korea, about 9 out of 10 people own a smartphone and the usage time is steadily increasing (Jeong et al., 2020). More than 2.23 billion people use Facebook monthly, and appear to share their thoughts on social issues and details of friends through this platform (Wiese et al., 2020). In addition, more than 90% of adults in the United States visit social media websites, and in the United Kingdom people spend an average of 136 min a day on social media; such people make social comparisons through their use of social network services (SNS) and their behavior is significantly influenced by these social comparisons (Oh et al., 2014).

The 2018 Digital Information Gap Survey Report published by the National Information Society Agency (NIA) comprehensively measured the level and characteristics of the information gap occurring in the digital environment of South Korea and found that, taking the level of the general public as 100, the level of digital informatization of people with disabilities was 74.6% as of 2018. This was higher than the average (68.9%) of people with disabilities, low-income, farmers, fishermen, and the elderly, which comprised the information-vulnerable groups in the digital information gap survey and was the second highest after the low-income group (86.8%). Even relative to other groups, this information gap for people with disabilities has been increasing every year (National Information Society Agency, 2019).

Quantitative growth and the narrowing of the gap in accessibility are the result of policy measures, such as the information and communication assistive device distribution project and development projects for people with disabilities who have difficulty accessing information due to physical and economic conditions (Baxter et al., 2012; Duplaga, 2017). The use of the Internet can provide opportunities for people with disabilities to enhance their independence, access online services such as e-banking and Internet shopping, and communicate with family and friends through e-mail or video conferencing. Through such ways, it can greatly improve their daily lives (D’Aubin, 2007; Duplaga, 2017). The Internet can be a viable way to promote social participation for people with disabilities (Raghavendra et al., 2013) who can use information and communication assistive devices to overcome their physical limitations and to engage in socio-economic activities to expand their rights and interests (Jin, 2013). Therefore, there is a need for an empirical study on how the use of digital smartphones affects the psychology of individuals, beyond the accessibility gap issue.

In recent years, with the global increase in smartphone use, studies on the relationship between this usage and emotional risk behaviors, such as depression, anxiety, and suicide-related behaviors, have been continually conducted (Lemola et al., 2015; Sueki, 2015; Twenge et al., 2018). Previous research on people with disabilities has confirmed that digital use influences life satisfaction and satisfaction with policies (Lee and Lee, 2018; Hwang, 2019). In a study that investigated the relationship between social media use and well-being in people with physical disabilities, it was reported that the higher the intensity of SNS use and online community use, the lower the level of depression through intervention tools and information support. It was reported that social media use played a positive role in building social support and strong psychological tendencies (Lee and Cho, 2019). In other words, there is a possibility that people with disabilities experience a positive impact on their lives through online activities.

Studies involving Internet use and individuals with visual impairments have generally focused on issues related to barriers to access and use (Williamson et al., 2001; Gerber, 2003). Among these barriers are the difficulties associated with accessibility, cost, and assistive technology; while the perceived benefits of using the Internet include access to new information, ability to use the Internet in the workplace, and improved social participation. However, the relationship between Internet use and the psychosocial well-being of individuals with visual impairments has not yet been investigated. Initially, visually impaired people could not use mobile phones for other purposes than making calls; however, with the advent of the iPhone in 2009 and the improvement of services such as the voice over function, people with visual impairments were able to expand their mobile phone usage beyond just making phone calls. However, studies related to the use of smartphones among people with visual impairment have not been actively conducted. Therefore, it is necessary to investigate the impact of smartphone use on the lives of people with visual impairment and its effect on negative emotions in order to obtain basic data to plan future mobile device-related research projects for this population. Accordingly, this study aims to investigate the relationship between the degree of use of smartphones and negative emotions in people with visual impairment.

## Materials and Methods

### Data

This study utilized the data from the 2016 Internet Overdependence Survey conducted by NIA from September to November 2016 in South Korea. The survey covered a sample of 24,386 people (100,000 households) aged between 3 and 69, who resided in 17 cities and provinces nationwide, with a resulting sampling error of ±0.63 at a 95% confidence level. This study analyzed a subset of the overall data, covering 30 people with visual impairments. For the survey, a trained professional investigator personally visited the sample households and wrote down the subject’s responses to each question. Table 1 summarizes the general characteristics of the participants.

**TABLE 1:** General characteristics of people with visual impairment.

**Category**	**Sub-category**	***n***	**%**	**At high risk**	**At potential risk**	**General user**
Sex	Male	18	60.0	2	1	15
	Female	12	40.0	0	2	10
Age	18–29	2	6.7	0	0	2
	30–49	18	60.0	1	3	14
	Above 50	10	33.3	1	0	9
Education level	Graduate middle school	5	16.7	0	0	5
	Graduate high school	8	26.7	2	0	6
	Above college	17	56.7	0	3	14
Income level^+^	Below 2,000	3	10.0	0	0	3
	2,000–3,999	9	30.0	1	1	7
	4,000–5,999	11	36.7	0	0	11
	6,000–7,999	4	13.3	1	1	2
	8,000–9,999	2	6.7	0	0	2
	Above 10,000	1	3.3	0	1	0
Employment	Yes	29	96.7	1	3	15
	No	1	3.3	1	0	10

*^+^Income levels in 10,000 South Korean won (USD 1 = KRW 1,126.50).*

### Measure

The purpose of smartphone use was measured by classifying it into information search, leisure, communication, and online transactions, and using a seven-point Likert scale. Information search comprised searching for information related to news, academic/business, education/learning, product/service, and traffic/location. Leisure was defined as the use of smartphones for games, adult content, movies, TV and video content, music, e-books, web cartoons and web novels, and speculative games (sports betting, online gambling, etc.). Communication covered the use of e-mail, messenger, and SNS. Lastly, online transactions covered product/service purchases and product/service sales.

Negative emotions can be defined in various ways. The questions related to negative emotions presented in representative emotion scales are as follows. The Positive and Negative Affect Schedule (PANAS; Watson et al., 1988) includes questions with negative emotions such as “frightened,” “hostile,” “nervous,” “sensitive,” “impatient,” “painful,” “broken heart,” “guilty,” and “shameful.” The Intensity and Time Affect Scale (ITAS; Diener et al., 1995) includes negative emotions such as “fear,” “anger,” “irritation,” “anxiety,” “worry,” “guilty,” “shame,” “disgusting,” “regret,” “sadness,” “loneliness,” “unhappy,” and “depression.” The Scale of Positive and Negative Experience (SPANE; Diener et al., 2009) includes negative emotions such as “sad,” “unpleasant,” “bad,” “negative,” “afraid,” and “angry.”

The emotional problems experienced by people with visual impairments were measured by a scale developed by National Information Society Agency (2019). This scale includes questions aimed at measuring emotions similar to the questions suggested by the emotion measurement tools used in previous research. The Cronbach’s α was reported as.880 (Bae, 2015). The emotional mood in the respondent’s daily life was assessed through several statements. The statement “I am depressed and de-motivated” was used to measure depression, “I am anxious” was used to measure anxiety, and “I am lonely” was used to measure loneliness. The responses to these statements were measured on a 4-point rating scale where one represented “I do not feel like this at all” and four represented “I feel this way often.”

### Statistical Analysis

Descriptive statistics were used to describe the characteristics of people with visual impairment. A one-sample Kolmogorov-Smirnov test was performed to check whether the continuous data used in our study was normally distributed, which is a prerequisite for conducting parameter estimations. Negative emotions such as depression, anxiety, and loneliness were not normally distributed (*p* < 0.05). In terms of smartphone use purposes, information search, leisure, and communication use were found to be normally distributed (*p* > 0.05) while the degree of online transaction utilization was not normally distributed (*p* < 0.05). In regression analysis, since both the independent and the dependent variables must follow normal distributions, this study conducted a partial least squares (PLS) regression analysis.

Interpretation of PLS analysis results can determine the magnitude of influence through variable importance in the projection (VIP). In general, if the VIP value is close to or exceeds 1, the coefficient of the variable can be considered statistically very significant. Even a VIP of 0.8 or higher would imply that the variable plays a significant role in estimating the causal relationship (Nash and Chaloud, 2011). This study used an empirical criterion that if the VIP value of a particular variable was less than 0.8 and the absolute value of all regression coefficients was very small (close to 0), it could be removed as a meaningless explanatory variable when extracting latent factors or estimating the causal relationship (Sawatsky et al., 2015). This study applied a PLS regression analysis using five potential factors, which are basic set values, to observe the change in explanatory power of the derived model according to a change in the number of potential factors. The explanatory power of the model can be determined by the cumulative y variance of the response variable Y. This explanatory power generally increases as variables are added until a specific point where the explanatory power decreases upon adding the next variable. This point is deemed as the cutoff and the number and specific factors included until this newly added variable are used to define the statistical characteristics of the model (Sawatsky et al., 2015).

All analyses were performed using SPSS 21.0 (SPSS Inc., Chicago, IL, United States) at a significance level of α = 0.05.

### Ethics Approval and Consent to Participate

This study did not require formal ethical approval under national laws. It used publicly available data from NIA, which did not contain any personally identifiable data. Ethical and governance approvals were granted by the Ministry of Science and ICT in South Korea. All participants gave written informed consent for participation before completing the survey.

## Results

### Explained Variance in Negative Emotion

Table 2 shows the effect of smartphone usage on the three negative emotions analyzed in this study.

**TABLE 2:** Explained variance in negative emotions.

**Negation emotion**	**Latent factors**	**Cumulative *X* variance**	**Cumulative *Y* variance (R-square)**	**Adjusted R-squared**
Depression	1	0.251	0.141	0.110
	2	0.379	**0.192**	0.132
	3	0.469	0.212	0.121
	4	0.631	0.214	0.088
	5	0.723	0.216	0.053
Anxiety	1	0.230	0.187	0.158
	2	0.387	**0.244**	0.188
	3	0.477	0.266	0.181
	4	0.615	0.271	0.154
	5	0.736	0.273	0.121
Loneliness	1	0.209	0.277	0.251
	2	0.370	0.332	0.283
	3	0.551	**0.345**	0.269
	4	0.679	0.349	0.245
	5	0.741	0.351	0.215

*Bold means the explanatory power of model.*

As shown in Table 2, the variance of the explanatory variables through each latent factor (Cumulative *X* Variance) was analyzed to explain 72.3% of the variance of all nine explanatory variables through five latent factors in depression, 73.6% in the case of anxiety, and 74.1% in the case of loneliness.

The explanatory power of the model can be determined by the cumulative y variance of the response variable Y, and the difference in explanatory power was reduced upon adding the third latent factor; hence, the explanatory power at this point and the number of potential factors (two) were determined as the statistical characteristics of the model. The explanatory power of the model constructed to determine the influence of smartphone use on the negative emotion of depression was therefore, 19.2%, which corresponds to the second potential factor. Similarly, the explanatory power of the model constructed for the effect of smartphone usage on anxiety and loneliness can be interpreted as 24.4 and 34.5%, which correspond to the second and third potential factors, respectively.

### The Results of PLS Regression Analysis

The models determining the effect of smartphone use on the negative emotions of depression and anxiety were analyzed based on the importance value of the second latent factor, while the model for loneliness was based on the importance value of third latent factor. Table 3 lists the resulting importance of the explanatory variables for each potential factor through the VIP values of the five potential factors.

**TABLE 3:** The PLS regression analysis results.

**Negative emotion**	**Variables**	**B**	**Latent factors**				
			**1**	**2**	**3**	**4**	**5**
Depression	Constant	2.616					
	Sex	0.047	0.478	0.536	0.665	0.663	0.665
	Age	−0.136	0.816	0.701	0.870	0.870	0.886
	Education level	−0.134	0.618	0.532	0.509	0.542	0.541
	Income	−0.080	0.597	0.514	0.584	0.583	0.584
	Information search	0.043	1.719	**1.489**	1.440	1.438	1.447
	Leisure	0.277	1.810	**1.694**	1.641	1.633	1.629
	Communication	−0.200	0.000	**1.347**	1.377	1.371	1.366
	Online transactions	0.027	0.369	0.353	0.336	0.386	0.394
Anxiety	Constant	2.635					
	Sex	−0.248	0.716	0.837	0.803	0.807	0.805
	Age	0.239	0.407	0.507	0.660	0.655	0.667
	Education level	−0.014	0.309	0.396	0.418	0.447	0.446
	Income	0.021	0.099	0.278	0.455	0.452	0.471
	Information search	−0.106	1.480	**1.297**	1.270	1.261	1.257
	Leisure	0.017	1.033	0.940	0.907	0.932	0.941
	Communication	−0.322	1.174	**1.608**	1.573	1.563	1.558
	Online transactions	0.105	0.190	0.711	0.704	0.700	0.701
Loneliness	Constant	0.864					
	Sex	0.301	0.867	0.815	0.823	0.824	0.828
	Age	0.228	0.165	0.416	0.499	0.498	0.505
	Education level	−0.043	0.561	0.587	0.675	0.671	0.670
	Income	0.266	1.003	1.160	**1.139**	1.132	1.130
	Information search	−0.012	0.904	0.912	0.902	0.932	0.943
	Leisure	0.232	1.158	1.058	**1.045**	1.039	1.037
	Communication	0.012	0.279	0.596	0.593	0.603	0.611
	Online transactions	−0.418	1.914	1.831	**1.824**	1.821	1.816

*Bold means variable importance in the projection.*

As a result of the PLS regression analysis, the most important factor influencing the negative emotion of depression was leisure with the highest importance (VIP) of 1.694. Information search (1.489) and communication (1.347) were other factors that were considered important as their VIP values were 1.0 or higher.

Regarding anxiety, leisure was estimated as an important factor with the highest importance (VIP) at 1.608. Based on a VIP value exceeding 1.0, other factors deemed important variables were information search (1.297) and communication (1.608). In addition, the variables with VIP values between 0.8 and 1.0 were sex (0.837) and leisure (0.940).

Lastly, in the case of loneliness, the variable that was determined to be the most important influencing factor was online transactions, with the highest importance (VIP) at 1.824. Leisure (1.045) and income (1.139) were other important factors with VIP values exceeding 1.0. In addition, the variables with VIP values ranging between 0.8 and 1.0 were gender (0.823) and information search (0.902).

## Discussion

To determine whether the use of smartphones affected negative emotions such as depression, anxiety, and loneliness in people with visual impairment, this study analyzed the data from the 2016 Internet Overdependence Survey of South Korea.

First, it was found that the use of smartphones for leisure, information search, and communication purposes by people with visual impairment significantly influenced their negative emotions. Regarding depression, the non-standardized coefficient of communication was negative (-) indicating that the more that smartphones were used for communication, the lower the negative emotion of depression. Second, the use of smartphones for information search and communication was associated with lower anxiety in people with visual impairment. Third, regarding the effect of the use of smartphones by people with visual impairment on the negative emotion of loneliness, it was found that income, leisure, and online transactions had significant effects. Among these, the coefficients for income and leisure were positive, indicating that the higher the income or the greater the use of smartphones for leisure, the greater was the negative emotion of loneliness. The coefficient of online transactions was negative indicating that the use of smartphones for online transactions was associated with lower loneliness levels.

Just as it cannot be denied that the Internet and the use of smartphones have made our lives more convenient than before, not all aspects of using smartphones have a positive impact (Demirci et al., 2015; Elhai et al., 2016). Studies that show that smartphone use is related to negative emotions such as depression and anxiety have been reported steadily. In particular, research results on the effect of problematic smartphone use have been reported (Rozgonjuk et al., 2018). Social smartphone use is negatively associated with anxiety and depression symptoms severity (Elhai et al., 2017). This indicates that using a smartphone for communication is associated with lower depression and anxiety. The aforementioned findings are consistent with the results of this study, which showed a negative relationship between the use of smartphones for communication and the negative emotions of depression and anxiety. A possible explanation for this negative relationship is that the proportion of people at risk of developing smartphone addiction among people with visual impairments (16.7%; National Information Society Agency, 2019) was lower than that of the total survey group (20.0%; National Information Society Agency, 2019). The use of online chat or instant messaging by people with visual impairment has been reported to have a positive effect on their psychological well-being while information retrieval has been reported to have a negative effect (Smedema and McKenzie, 2010). The social use of smartphones and the low ratio of addiction associated with their usage in people with visual impairment might have an effect on the negative relation between communication and depression and anxiety.

It has been argued that the use of social networks has a negative impact on emotions. An excessive use of Facebook not only caused a phenomenon of “Facebook depression” among teenagers (O’Keeffe and Clarke-Pearson, 2011), but the literature also uncovered a high correlation between the frequent use of Facebook and depression (Pantic et al., 2012; Oh et al., 2014). This is because many people participate in social comparison behaviors through social network sites like Facebook, and their behaviors are significantly influenced by such social comparisons (Zhang and Centola, 2018). SNS have also been shown to negatively affect an individual’s emotional state (Sagioglou and Greitemeyer, 2014). In other words, while communication through direct conversation on Facebook increases bonding social capital and reduces loneliness, the longer one uses Facebook without any interaction, the lesser the social capital and greater the loneliness (Burke et al., 2010). This is because information such as vacation photos of other people on Facebook induces envy in viewers, which in turn negatively affects life satisfaction (Krasnova et al., 2013). By replacing strong connections with weak connections through social media use and reducing direct face-to-face contact with people close to us, we become unhappy by creating emotions such as “alone while together” or “solitude in a crowd.” In the case of people who use the Internet for communication, it was reported that the depression score decreased at the follow-up after 6 months (Bessière et al., 2008). In addition, using the Internet for communication purposes is a predictor of low depression while using the Internet for purposes other than communication has been reported to predict high levels of depression and social anger (Selfhout et al., 2009). The results of this study, which showed that the use of smartphones for leisure and information search was positively related with the negative emotions of depression and loneliness among people with visual impairment, are consistent with these previous studies.

Information search can be motivated by the aim of reducing uncertainty when a problem occurs; it has been reported that provision of information relieves anxiety in situations such as when facing surgery (Sjöling et al., 2003). Moreover, the pursuit of security through information search can be an important issue in online health-related searches (Starcevic and Berle, 2013). Since previous studies are related to health-related information retrieval and hepatic anxiety, it is difficult to directly compare them with the results of this study; in this sense, this study can be said to be different. Therefore, further research on the relationship between the use of smartphones for information retrieval and anxiety is necessary.

Partial least squares regression analysis can capture the influence of factors regardless of the sample size. Moreover, since each independent variable is modeled using least squares, the analysis is free from the problem of multicollinearity. In addition, it has the advantage that the predictive power is superior to the principal component regression analysis because the dependent variable and the independent variable are considered simultaneously when the principal component is extracted. For all its methodological advantages, this study conducted a PLS regression analysis, using panel data with systematic sampling.

Nonetheless, this study has several limitations. First, the sample size was relatively small. Future research should increase the number of participants so that the effects of degree of smartphones use on negative emotions in people with visual impairment can be verified through various methods such as path analysis. Second, the questionnaire to measure emotional mood is too simple to capture all emotional well-being. The information available for this study was limited to what was provided by the panel data. In future studies, it is necessary to obtain more detailed information about relation between the degree of use of smartphones and negative emotions in people with visual impairment. Third, the data analyzed were from 2016. Although this is the most recent data on Internet use and negative emotions from the national information gap survey, as a result of improvements on the usage restrictions since then, the present impact on negative emotions may be less. Therefore, research is needed to assert the current relationship between the use of smartphone’s features and negative emotions.

## Data Availability Statement

Publicly available datasets were analyzed in this study. This data can be found here: https://eng.nia.or.kr/site/nia_eng/main.do.

## Ethics Statement

Ethical review and approval was not required for the study on human participants in accordance with the local legislation and institutional requirements. Written informed consent to participate in this study was provided by the participants’ legal guardian/next of kin.

## Author Contributions

E-YP contributed to project administration, data analysis, and writing.

## Conflict of Interest

The author declares that the research was conducted in the absence of any commercial or financial relationships that could be construed as a potential conflict of interest.
